# Social Isolation and Loneliness in People Living With Chronic Kidney Disease and Kidney Failure: A Mixed Method Systematic Review

**DOI:** 10.1111/jorc.70049

**Published:** 2026-02-07

**Authors:** Amanda L. McKie, Paul J. Buttel

**Affiliations:** ^1^ University of Canberra Canberra Australian Capital Territory Australia; ^2^ Department of Health Disability and Ageing Canberra Australian Capital Territory Australia

**Keywords:** chronic kidney disease, kidney failure, loneliness, social isolation, systematic review

## Abstract

**Background:**

People living with chronic kidney disease and kidney failure experience a wide range of challenging burdens, as many try to manage their condition effectively. Social isolation and loneliness are commonly associated with chronic kidney disease and kidney failure, and the restrictions of their disease make establishing social connections difficult. The impact of social isolation and loneliness can negatively affect a person's health and wellbeing in many ways.

**Objective:**

To conduct a mixed method systematic review of studies about social isolation and loneliness in people with chronic kidney disease and kidney failure.

**Design:**

Using the PRISMA guidelines, six databases (Medline, CINAHL, Cochrane Library, PsycINFO, Scopus and Google Scholar) were comprehensively searched using keywords from June 2005 to August 2025. Studies were quality assessed.

**Results:**

Seven studies met the eligibility criteria (two quantitative and five qualitative research design). Four common factors emerged from the review of studies that included: coping, support, psychological outcomes and impacts that affect everyday life and daily routine.

**Conclusion:**

Social isolation and loneliness impact people living with chronic kidney disease and kidney failure in many ways across all areas of their lives. Negative emotions arose that made people question the point of continuing treatment as others experienced elevated levels of anxiety and depression associated with social isolation and loneliness. Support services and early interventions need to be considered to better manage social isolation and loneliness for people living with this chronic health condition.

## 1 Introduction

Currently, 11% of Australians aged 18 and over are affected by chronic kidney disease (CKD) (Australian Institute of Health and Welfare AIHW 2024) while, many others remain largely unaware and undiagnosed within the early grades of CKD (Kidney Health Australia KHA 2024). Chronic kidney disease includes irreversible damage to kidney function that has been sustained for at least 3 months in duration (Kalantar‐Zadeh et al. 2021). Many people do not realise they have CKD due to the asymptomatic nature of the condition (Australian Institute of Health and Welfare [AIHW] 2024). There are five grades of CKD (previously known as stages), grade one is where signs and symptoms remain almost unnoticed and difficult to detect (Kidney Health Australia [KHA] 2024). The last grade of CKD is Grade 5 kidney failure (formerly known as end‐stage kidney disease), where kidney function is affected so severely that kidney replacement therapy needs to be considered to sustain life (KHA 2024; Levey et al. 2020). Kidney replacement therapy (KRT) typically includes peritoneal dialysis (PD) or haemodialysis (HD), kidney transplantation (KT) or supportive care measures (American Diabetes Association Professional Practice Committee 2024). Chronic kidney disease and kidney failure are the fastest growing cause of death globally, further highlighting the health burden internationally (World Health Organization [WHO] 2025).

People living with CKD and kidney failure experience many burdens that impact all areas of their lives (Almutary et al. 2016; Bikbov et al. 2020; Yapa et al. 2024). Social isolation has been well documented in people with chronic and complex conditions (Margono 2024; Riazuelo 2021) and linked to high levels of anxiety and depression and low levels of quality of life (Iovino et al. 2023). The effects of social isolation were exacerbated by the COVID‐19 pandemic, as postponed medical appointments and delayed treatment resulted in people feeling unsupported and alone (Iovino et al. 2023). Whereas feelings of loneliness can restrict social interaction and can lead to feelings of hopelessness (Margono 2024).

The constraints of HD and PD treatment have a profound social impact, often leading individuals to report feelings of loneliness (McKie et al. 2023; Sharma et al. 2019). The demands of HD and PD make it challenging for people to plan and organise holidays or social events, as treatment schedules dominate much of their lives (Lee et al. 2007; McKie et al. 2023). Additionally, the restrictions imposed by KRT, may cause many individuals to deliberately withdraw from social activities. This withdrawal is frequently attributed to the physical effects of treatment, such as fatigue and pain, as well as the fear of friends and family members seeing them unwell (De Silva et al. 2021; Lee et al. 2021; McKie et al. 2023).

Social isolation is all too common in older adults with chronic health conditions as the lack of physical mobility may restrict their ability to be mobile and interact socially with others (Gerlach et al. 2024; Margono 2024) however, people of all ages living with chronic conditions have identified health impacts from social isolation (Miao et al. 2025; Thompson et al. 2025). Social isolation can be defined as the lack of social connection including friends, family or people within the community (Gerlach et al. 2024; Margono 2024). Having a lack of social connection due to isolation may lead to significant vulnerability in health, as some people struggle with their health concerns alone (Motillon‐Toudic et al. 2022; Holt‐Lunstad and Steptoe 2022). Interestingly, some people can be surrounded by others but still experience feelings of loneliness (Gerlach et al. 2024; Goldman et al. 2024). However, people who are experiencing isolation do not necessarily feel lonely and vice versa (Gerlach et al. 2024; Goldman et al. 2024).

Loneliness is considered an unpleasant emotional state where a person feels in need of others (Fakoya et al. 2020; Hwang et al. 2020). As a subjective negative emotion, loneliness can also be defined as a lack of social connection in the absence of companionship (Fakoya et al. 2020; Margono 2024). Some definitions of loneliness describe how people's social needs are not being met by the quantity and quality of their existing social relationships (Barjaková et al. 2023; Luhmann et al. 2023). Feelings of loneliness can be influenced by the environment, demographic factors and current world events (such as Covid‐19) that may be beyond the individual's level of control (Barjaková et al. 2023; Luhmann et al. 2023). Nevertheless, both social isolation and loneliness can impact a person wellbeing and way of life.

Social isolation and loneliness often go together for some people (Mushtaq et al. 2023) coupled with trying to effectively manage a chronic condition such as CKD, can prove challenging (Keskin et al. 2019; McKie et al. 2023). Previous research into social isolation and loneliness has identified that these concepts are often the outcome of unmet supportive care needs and inadequate coping strategies (McKie et al. 2023; Mizumoto et al. 2024). Therefore, this timely review aims to identify the impact of social isolation and loneliness in people living with CKD and kidney failure.

This will be the first systematic review that will critically review and combine qualitative and quantitative studies to determine the effects of social isolation and loneliness, specifically in adults living with CKD and kidney failure. The review will aim to answer the following questions:

1. What is known about social isolation and loneliness of people affected by kidney failure requiring?
2. What is the impact of social isolation and loneliness on people living with kidney failure?

## 2 Methods

The mixed methods review was conducted according to the Preferred Reporting Items for Systematic Reviews and Meta‐Analyses (PRISMA) guidelines (Page et al. 2021), incorporating both quantitative and qualitative studies. A convergent design was selected whereby both quantitative and qualitative studies were analysed separately before converging into an integrated synthesis of evidence. The approach was selected as it allows different aspects of the same concept to be explored, specifically how social isolation and loneliness impact all areas of a person's life. The review aimed to capture the quantitative studies to explore the impact of social isolation and loneliness and then summarise the qualitative studies that identify the lived experiences of the phenomenon. The studies were analysed separately before being integrated into a narrative synthesis of mixed method findings. The systematic review followed the registered a priori protocol available from PROSPERO (CDR42024617854).

### 2.1 Inclusion Criteria

Quantitative studies were eligible for inclusion if they reported on social isolation and loneliness and broader outcomes. All qualitative studies, irrespective of research design, that include adults affected by CKD and kidney failure regardless of KRT type. Only studies that are in the English language and in a peer review journal (see Table 1).

**Table 1:** Inclusion and exclusion criteria.

Selection criteria	Inclusion criteria	Exclusion criteria
Language	English	Non‐English
Dates	Publication from November 2005 to August 2025	Publications before 2004
Study types	Quantitative research, qualitative research, mixed methods and systematic reviews	Conference abstracts, reports, case studies, news articles, editorials
Topics	CKD, kidney failure, loneliness, lonely, isolation, social isolation	Kidney cancer, AKI

Abbreviations: AKI, acute kidney injury; CKD, chronic kidney disease.

### 2.2 Exclusion Criteria

Case reports, conference abstracts, commentaries, editorials, news articles or studies without data to address the research questions, and unpublished primary studies.

### 2.3 Types of Participants

All adults (≥ 18 years) living with CKD or kidney failure, irrespective of KRT modality.

#### 2.3.1 Search Strategy

The Medline, CINAHL, Cochrane Library, PsycINFO, Scopus, and Google Scholar were searched for all relevant studies. Relevant systematic reviews were also scrutinised for potentially related studies for screening. A wide range of keywords and subject headings was used to increase sensitivity and inclusiveness, and the searches included from June 2005 to August 2025. The search dates were selected because of the emerging trend of research about the impact of social isolation and loneliness in older people living with chronic conditions (Hoang et al. 2022). See Table 2 for full record database searches.

**Table 2:** Search strategy (CINAHL).

1.	“Loneliness” OR “lonely” OR “alone”	12,269
2.	“Social isolation” OR “social alone”	17,137
3.	“End‐stage kidney disease” OR “ESKD” OR “end stage renal disease” OR “ESRD” OR “end‐stage kidney failure” OR “ESKF” OR “kidney failure” OR “chronic kidney disease” OR “CKD” OR “renal replacement therapies” OR “RRT” OR “peritoneal dialysis” OR “haemodialysis” OR “haemodialysis” OR “HD” OR “dialysis” OR “renal” NOT “predialysis” NOT “transplant”	45,076
4.	1 AND 2 AND 3 AND 3 AND 4	8
5.	Limiters‐English	8
6.	Limiters‐Date of Publication 2005, to August 2025	8
7.	5 AND 6 AND 7 AND Journal article OR Review	8

#### 2.3.2 Study Selection

The study selection process was conducted in the Covidence systematic review software. After de‐duplication, two reviewer authors independently screened the titles and abstracts of the identified studies for eligibility using Covidence systematic review software. The full text of all potentially eligible studies was retrieved and then screened independently by two review authors, and any conflicts were resolved by discussion. The reference list of the eligible studies was also screened to identify any further studies.

#### 2.3.3 Critical Appraisal

Using the Mixed Methods Appraisal Tool (MMAT) (Hong et al. 2018) two reviewers conducted the critical appraisal of the individual studies. The quality assessment of the included studies was determined by the MMAT tool that includes five questions about each study design and any differences in answers were resolved by discussion among the reviewers (see Supporting Information: Table S1).

Two of the studies (Asti et al. 2006; Saedi et al. 2019) were assessed using the quantitative methodology criteria of the MMAT tool (Hong et al. 2018) and were rated reasonable quality however, the risk of nonresponse bias was unclear. The other five qualitative studies (Diao et al. 2023; Jeong et al. 2025; Malo et al. 2022; Sluiter et al. 2024; Zou et al. 2024) were assessed with the qualitative methodological quality criteria, and all were of high quality with clear research questions, data collection, findings, results and coherence between sections.

#### 2.3.4 Data Extraction and Synthesis

Data was extracted based on the JBI Mixed Methods Data Extraction Form, including the study type, sample, setting, participant characteristics, CKD grade, KRT, results and key findings (see Table 3). The integrated results synthesis is presented in Table 4 according to the Mixed Method Systematic Reviews (MMSR) guidelines (Stern et al. 2020). A convergent segregated approach was undertaken to combine the qualitative and quantitative findings to answer the review question about the impact and experiences of social isolation and loneliness in people with CKD and kidney failure irrespective of KRT (Stern et al. 2020).

**Table 3:** Summary of studies included in the review.

Author, country	Methodology/methods for data collection and analysis	Sample (patients and/or caregivers)	Setting	Participants characteristics (M, SD, %, ages in years, range)	CKD treatment	Results	Outcome/phenomena of interest
Asti et al. (2006) Turkey	Quantitative: Descriptive correlational. Data collection: UCLA loneliness scale, Beck depression inventory perceived social support scale. Analysis: Descriptive, *t*‐tests, Pearson correlations.	*n* = 130 *n* = 65 *n* = 65 caregivers	Renal Clinics	Female: 26% Male: 74% Age 44.60 ± 17.22 Caregivers: 43.90 ± 8.52	CAPD: 65 people	No significant relationship between loneliness and depression in patients. Above average levels of social support	Loneliness, depression and social support
Diao et al. (2023) China	Qualitative: Phenomenology. Data collection: Semi‐structured interviews. Analysis: Content analysis	*n* = 12	On‐line	Female: *n* = 6 Male: *n* = 6 Ages: 19–60 years	PD	Three main themes and 12 sub themes	SI is common for many receiving PD treatment
Jeong et al. (2025) South Korea	Qualitative: Phenomenology. Data collection: Semi‐structured and open‐ended interviews. Analysis: Giorgi's analysis method.	*n* = 11	Renal clinic	Female: *n* = 7 Male: *n* = 4 Ages: 45–62 years	HD	Seven themes were derived	The experiences of loneliness. A sense of loss due to the repetitious nature of HD treatment
Malo et al. (2022) Canada	Qualitative: Exploratory. Data collection: semi‐structed interviews. Analysis: Thematic content analysis.	*n* = 22	Home via telephone or video conference	Female: *n* = 9 Male: *n* = 13 Ages: 30–90 years	HD	Three main themes	The impact of the Covid‐19 pandemic on relationships caused feelings of isolation from loved ones
Saedi et al. (2019) Iran	Quantitative: Cross sectional. Data collection: Questionnaire, loneliness cale, social isolation form, IIEF, FSFI. Analysis: Descriptive, Spearman's correlation.	*n* = 132	Renal clinics	Female: *n* = 77 Male: *n* = 53 Ages: 54.26 ± 9.91	HD	Low levels of loneliness. No statistical relationship between loneliness, social isolation, sexual function and self‐esteem	Interventions are required to improve and manage social isolation and loneliness in people receiving HD is required
Sluiter et al. (2024) Australia	Qualitative: Descriptive Data collection: Analysis: Thematic content analysis	*n* = 1009 Caregivers *N* = 260 *n* = 8 identified as a patient and a caregiver	Renal clinics	Female: *n* = 528 Male: *n* = 481 Ages: 18–80 years	HD, PD and TX	Themes emerged about restrictions, fending for oneself, diminished role, vulnerable to the environment, self‐blame and feeling excluded	The perspectives and experiences of social isolation and loneliness
Zou et al. (2024) China	Qualitative: Descriptive Data collection: Face‐to‐face semi structured interviews Analysis: Deductive content	*n* = 15	Renal clinic	Gender of participants not specified. Ages: 18–80 years	HD	Three main themes	Multi‐level interventions are required to adequately manage social isolation

Abbreviations: CKD, chronic kidney disease; CAPD, continuous ambulatory peritoneal dialysis; IIEF, International Index Of Erectile Function; FSFI, Female Sexual Function index; HD, haemodialysis; SD, standard deviation; Tx, transplantation.

**Table 4:** Key results of included studies.

Author	*n*	Outcomes	Results	Key findings
Asti et al. (2006)	*n* = 130 *n* = 65 *n* = 65 caregivers	Levels of loneliness, depression, and social support	No significant relationship between loneliness and depression in patients. Above average levels of social support were reported.	Only a few people experienced loneliness, and many others rated their level of social support as ‘above average’
Diao et al. (2023)	*n* = 12	Experiences of social isolation	Three main themes and 12 sub‐themes were derived. The first theme is: Dialysis treatment stimulates problems with social isolation with four sub themes: low self‐esteem and sensitivity, fear and concern within the unknown nature of CKD, self‐isolation and avoidance and alienation by others. Second theme includes: Struggles to escape the effects of social isolation with four sub themes of: Increasing cognitive and behaviour management, seeking support within the family, compromise and growth, reinventing the value of life. The third theme included: Multiple obstacles exacerbate the plight of social isolation with four sub‐themes that are: Shackles of over protection, agony of public misunderstanding burden of treatment expenditure and deficiencies in support systems.	Social isolation is common for many receiving PD treatment as the multiple daily dialysis sessions may contribute to the amount of time and distance that people can dedicate to socialising. *“Decades of buddies, from childhood to growing up, do not contact. About dinner and drink, I cannot eat high potassium, phosphorus food, cannot drink” (patient receiving PD)*. *“My partner ran away, we were all set to be married, as soon as she heard I had this disease, her family said ‘nothing to marry' here” (male patient receiving PD)*.
Jeong et al. (2025)	*n* = 11	Experiences of loneliness	Seven main themes were derived from the study The first them included: The loneliness felt in a life tied to HD like shackles. The second theme was the sorrow and loneliness of my irretrievable life. The third theme was the helplessness in death and isolation at the edge of life. The fourth theme included: Living everyday wrapped in solitude. The fifth theme were the complex emotions and alienation within the family. The sixth theme was the lonely life in the shadow of illness and societal prejudice. The seventh theme included struggling to break free from the abyss of loneliness.	People felt loneliness due to the social isolation and limitations of HD treatment. The sense of loss due to the repetition of HD treatment meant that many were ‘missing out’ on life events. *“In the past, I wanted to live comfortably or in a quiet countryside, but now I'm receiving HD, so actually I have to continue to live near a hospital, which makes my life restricted” (patient receiving HD)*. *“To be honest, I don't look like a sick person when I go out, but if people find out, they will feel sorry for me. I don't like that. I'm the only one who knows how lonely this is…” (patient receiving HD)*.
Malo et al. (2022)	*n* = 22	Experiences and feelings of loneliness	Three main themes and 11 sub themes were evident. The first theme included: the effects of the COVID‐19 pandemic on HD care and routine with four sub themes that are: effects on HD care, change in routine, change in routine regarding transportation and switching from in‐centre dialysis to home HD. The second theme was the effects of COVID‐19 infection risk and mitigation measures, and the 5 subthemes were: concerns relating to their own infection risk, risk management, appropriate measures, prohibition of visitors, and fear of infecting their loved ones. The third main theme were: Effect of COVID‐10 pandemic on relationships and included 2 sub themes. Interactions with health care workers and COVID‐19 pandemic and isolation from loved ones.	The impact of the Covid‐19 pandemic on relationships caused negative feelings that disrupted their normal routine and caused feelings of loneliness from the social isolation away from loved ones. *“I stay away, I talk on the phone, but there's no physical interaction at all (patient receiving HD)*.” *“Its obvious we all feel really isolated” (patient receiving HD)*.
Saedi et al. (2019)	*n* = 132	Levels of social isolation, loneliness and sexual dysfunction	People receiving HD reported low levels of loneliness. Social isolation levels were moderate. No statistical relationship between loneliness levels, and social isolation	The results indicate that people living with HD have adapted to the social limitations associated with HD. Low levels of loneliness reported. On going interventions are required to improve and manage social isolation and loneliness in people receiving HD.
Sluiter et al. (2024)	*n* = 1009	Experiences of social isolation and loneliness	Themes emerged that were about the restriction of the disease, fending for oneself in health care, diminished societal role, vulnerable in the external environment, feeling excluded and undermining self esteem	The burden of CKD impact not only individuals but their people and friends within their social unit. The fear of further infections restricts social activity and further isolated people living with CKD and led to feelings of loneliness that affected their wellbeing. *“I can't go abroad. I have relatives all over the place, but I can't attend any function” (patient receiving HD)*. *“Seeing everybody going, working, and I'm at home and staying there doing my dialysis (patient receiving PD)”* *“You can't just jump in the car and go” (patient receiving HD)*.
Zou et al. (2023)	*n* = 15	To understand coping strategies in the context of social isolation	Three main themes were identified. The first theme was: The prerequisites for coping with social isolation. The second theme were to maintain the bond between coping and social isolation. The third theme included the results of coping with social isolation.	Multi‐level interventions are required to adequately manage social isolation that is in collaboration with the family, hospital and community to better support people. *“Dialysis is my privacy; I do not need to tell anyone” (patient receiving HD)*. *“I think of it as a job, and I only have to work thrice a week, 4 h day, and my job is to lie down and talk to you in my sleep, and my pay is to be healthy” (patient receiving HD)*.

Abbreviations: CKD, chronic kidney disease; CAPD, continuous ambulatory peritoneal dialysis; IIEF, International Index of Erectile Function; FSFI, Female Sexual Function index; HD, haemodialysis; PD, peritoneal dialysis.

## 3 Results

**Figure 1:**
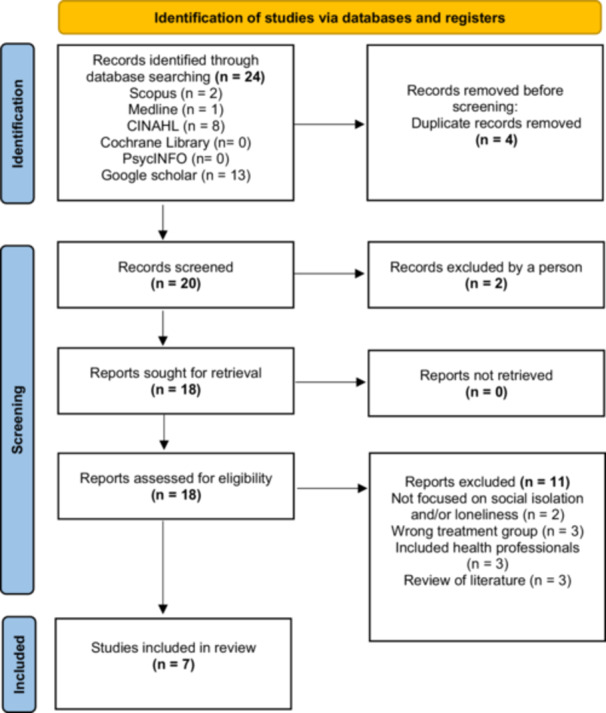
PRISMA 2020 flow diagram. The Prisma flow diagram (Page et al. 2021) represents the screening process of the review. Twenty four studies were identified through database screening and seven were included in the review.

A comprehensive search of six databases revealed only 24 records, after which 17 were removed, leaving seven in the final review (see Figure 1). Some of the studies removed did not focus on social isolation and/or loneliness specifically and some studies includes health professionals as the participants. The countries that were represented within the studies were: Australia (*n* = 1), Canada (*n* = 1), China (*n* = 2), Iran (*n* = 1), South Korea (*n* = 1) and Turkey (*n* = 1). The sample sizes across the studies ranged from 11 to 1009 and included KRT modalities such as HD and peritoneal dialysis. The search yielded no studies that specified ‘supportive care’ CKD.

**Figure 2:**
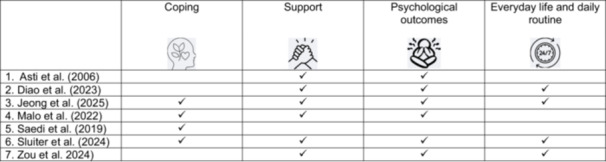
The four common areas identified across the included studies. identifies the four common aspects within the studies. Coping, support, psychological outcomes, and everyday life and routine where identified. The figure shows the studies that have the common aspects. Two of the studies identified all four aspects.

A total of seven studies published between 2005 and 2025 were included in the review. Four studies were qualitative research of which two used a phenomenology design (Diao et al. 2023; Jeong et al. 2025). One study used an exploratory design (Malo et al. 2022) and the others employed a descriptive qualitative approach (Asti et al. 2006; Sluiter et al. 2024). One study used a cross‐sectional design (Saedi et al. (2019) and the other was a descriptive correlational research design (Asti et al. (2006). Four common areas were identified across the seven studies that were (1) coping mechanism used to address loneliness and social isolation (2) support, (3) psychological outcomes of loneliness and social isolation and (4) everyday life and daily routine (see Figure 2).

### 3.1 Coping Mechanism

The utilisation of positive and negative coping mechanisms was reported in four of the studies (Jeong et al. 2025; Malo et al. 2022; Saedi et al. 2019; Sluiter et al. 2024). Jeoung et al. (2025) found that some people living with CKD tried to convince themselves that loneliness is normal and that being alone was a natural part of their disease that must be endured. While others with kidney failure (and receiving HD) often masked their real feelings of physical pain to family and friends as they thought if family and friends knew about their pain, they would be further isolated and not socially included (Jeong et al. 2025; Saedi et al. 2019; Sluiter et al. 2024). These types of coping mechanisms further compounded their feelings of loneliness and isolation in managing their disease effectively.

Conversely, positive coping mechanisms using social media were reported in two qualitative studies (Jeong et al. 2025; Malo et al. 2022). Using FaceTime to connect with others was regularly utilised along with recreational activities (using devices) such as listening to music, reading and fitness apps were employed by many participants to combat the feelings of loneliness and help promote activity and movement (Jeong et al. 2025; Malo et al. 2022). Saedi et al. (2019) also found that people adapted quickly to the conditions of their disease and accepted the social limitations, as Saedi et al. (2019) reported low levels of loneliness and only moderate levels of social isolation in people receiving HD.

### 3.2 Support

The importance of support from family and friends was reported in six of the studies (Asti et al. 2006; Diao et al. 2023; Jeong et al. 2025; Malo et al. 2022; Sluiter et al. 2024; Zou et al. 2024). Diao et al. (2023). Jeoung et al (2025) and Zou et al. (2024) indicated that support from family and friends was invaluable and was often provided in different ways, such as phone conversations, offering transport when needed and helping manage the household shopping.

Many people living with CKD had a deep appreciation of the support provided to them from family members to ensure they were cared for, felt important in the family unit and not alone (Zou et al. 2024). According to Asti et al. (2006), the level of perceived social support from family and friends was slightly different at times, as support from family was higher than support from friends. Conversely, the lack of support from healthcare services often left people feeling alone and unsure (Diao et al. 2023; Sluiter et al. 2024). As many people reported difficulties in accessing services and lacked information about how to seek support from the health system (Diao et al. 2023; Sluiter et al. 2024), which further isolated people trying to manage CKD effectively.

### 3.3 Psychological Outcomes

Psychological outcomes such as anxiety and depression associated with loneliness and isolation were identified in six of the studies (Asti et al. 2006; Diao et al. 2023; Jeong et al. 2025; Malo et al. 2022; Sluiter et al. 2024; Zou et al. 2024). Many people living with CKD, experience negative moods and bouts of depression in trying to cope with the loneliness associated with their disease. Negative moods and emotions had damaging effects for some people living with CKD, as they wanted to stay away from others, felt like a burden to others and worthless as a person in the community (Diao et al. 2023; Sluiter et al. 2024). Negative views associated with comparing oneself to others who are physically healthy and the thoughts of discontinuation in KRT further compounded feelings of loneliness and isolation (Jeong et al. 2025; Zou et al. 2024)

The physical changes and limitations from CKD made some people feel sad, helpless, and unsure where to seek help (Diao et al. 2023; Jeong et al. 2025; Sluiter et al. 2024). Physical changes such as weight gain, food limitation, decline in physical strength and being covered in tubing and lines (from PD and HD), further compounded feelings of sadness and depression and made it difficult to socially connect with others (Diao et al. 2023; Jeong et al. 2025). Kidney transplantation posed challenges for some people, particularly regarding weight gain, which significantly impacted their quality of life and ability to be confident when socialising (Sluiter et al. 2024). The alterations to self‐esteem were clear in many studies as people experienced fear in managing their condition effectively and lacked confidence in living alone with CKD (Diao et al. 2023; Jeong et al. 2025; Malo et al. 2022; Sluiter et al. 2024; Zou et al. 2024).

Staying within a ‘closed’ environment (such as an isolation room), and being unable to go outside during treatment compounded with the fear of contracting infections, further isolated some people (Diao et al. 2023; Sluiter et al. 2024). Feelings of isolation and loneliness were exacerbated by global events, such as the COVID‐19 pandemic. Some people stated that loneliness was always a problem but became worse with the worldwide pandemic in 2022, and mandated isolations imposed to limit the spread of COVID‐19 did not help any further in managing feelings of loneliness (Malo et al. 2022; Sluiter et al. 2024).

Many studies reported depressive symptoms such as anxiety, depression and altered moods due to social isolation and loneliness (Diao et al. 2023; Jeong et al. 2025; Malo et al. 2022; Sluiter et al. 2024; Zou et al. 2024). Interestingly, Asti et al. (2006) found the opposite, only a quarter of participants reported depressive symptoms that stemmed from loneliness and no significant relationship was found between loneliness and depression in people with CKD (Asti et al. 2006). Asti et al. (2006) also discovered that people are likely to experience lower levels of depression if they are socially supported by friends and family.

### 3.4 Everyday Life and Daily Routine

Four of the studies included aspects of the everyday life and the routine of KRT for people living with CKD and kidney failure. Many had a strong appreciation for life and their family members' presence, which subsided feelings of loneliness and isolation in some cases (Diao et al. 2023; Jeong et al. 2025). Zou et al. (2024) and Sluiter et al. (2024) found that people living with CKD are very attached and conditioned to their daily routine, and disruptions can impact their everyday routine.

Some people embraced loneliness as part of their disease and perceived that it must be endured alone (Jeoung et al. 2025), others accepted CKD and made attempts to normalise their life through ongoing education and recreational activities such as tertiary education and outdoor sports (Diao et al. 2023). Similarly, people consider HD treatment to be like that of a job, a routine activity that needs to be completed to continue living (Zou et al. 2024).

## 4 Discussion

The review aimed to identify what is known about social isolation and loneliness in people living with CKD and kidney failure irrespective of KRT. The impact of social isolation and loneliness is debilitating and impacts all areas of their life. The issue of social isolation and loneliness has also been raised as a health concern for different age groups.

Within young people, social isolation and loneliness were associated with mental health problems and somatic conditions such as pain and breathlessness (Hämmig 2019; Thompson et al. 2025; Christiansen et al. 2021). As routine assessments of young people tend not to include identifying levels of social isolation and loneliness, it is often overlooked as current research focuses on social isolation and loneliness in older adults (Hämmig 2019; Thompson et al. 2025). According to Christiansen et al. (2021) and Miao et al. (2025) targeted prevention and early intervention are required to address social isolation and loneliness, regardless of the age group, as it has negative consequences and impacts a person's level of health and wellbeing.

There is ample evidence about the health impact of social isolation and loneliness, even before the COVID‐19 pandemic (Goldman et al. 2024; O'Sullivan et al.^ 2021^; Wu 2020) and although many different scales are available to report and identify social isolation and loneliness, there is very little consensus about which ones should be primarily used (Manera et al. 2022; Pomeroy et al. 2023). A consistent approach to measuring social isolation and loneliness in people with chronic conditions may provide an opportunity to offer early intervention and support services before health and wellbeing are affected (Hoang et al. 2022; Williams et al. 2021).

Two of the studies (Asti et al. 2006; Sluiter et al. 2024) also included caregivers as participants in their investigations of social isolation and loneliness. Caregivers of people with CKD experience a high level of burden, which may lead to burnout if adequate support is not provided (Sluiter et al. 2024; Shankar et al. 2025). Some caregivers reported that their loved ones had little interest in changing routines or socialising at different places due to fear of something going wrong, further contributing to caregivers' feelings of social isolation and loneliness (Asti et al. 2006; Sluiter et al. 2024). The emotional burden of caregiving for someone with CKD is well documented (Chu et al. 2023; Hovadick et al. 2021; Shankar et al. 2025), but much of the literature focuses on caregivers of people with CKD receiving HD, with less attention given to other modalities such as supportive care and PD (Driehuis et al. 2025; Bártolo et al. 2022).

Some studies discuss the importance of coping strategies in managing social isolation and loneliness when living with chronic conditions such as chronic respiratory disease and other chronic health conditions (Suen et al. 2023; Van Wilder et al. 2021). Providing practical workshops, therapy and information sessions about how to develop effective coping strategies to manage feelings of isolation and loneliness may lead to better self‐efficacy for some people living with chronic conditions such as CKD (Hui Joo et al. 2025; McKie and Gaida 2022; Paterson et al. 2024).

The restrictions associated with living with CKD and kidney failure is well known as KRT often restricts their social activities and promotes isolation for some (Kirkeskov et al. 2021; McKie and Gaida 2022; Sharma et al. 2019). For young people living with kidney failure, there is often a social strain on friendships and connections, as they face challenges around disclosing their illness, arising concerns about the potential impact on their relationships (Kerklaan et al. 2020; Paterson et al. 2024; Rupp et al. 2021). Conversely, attending the HD centre for KRT was an opportunity for some to socially connect with renal staff and other people living with CKD and kidney failure (Nilsson 2019; Senteio and Ackerman 2021). The importance of connecting people with others who are living with the disease can provide comfort in knowing they are not alone. The ability to attend renal clinics in person lessens the burden of social isolation. However, support from renal healthcare professionals needs to be more forthcoming (Shahgholian and Yousefi 2018; Sutherland et al. 2021).

### 4.1 Strengths and Limitations

This systematic review has several strengths. First, the review followed a rigorous process and included both quantitative and qualitative studies. The qualitative studies included allowed the concepts of social isolation and loneliness to be thoroughly explored. The review also included all people with CKD and kidney failure irrespective of KRT. However, only studies within the last 20 years were included in the review and this may have aided in the small number of studies found. Another limitation is that only studies published in the English language were included and some studies with a different cultural viewpoint about social isolation and loneliness may have been excluded as a result.

### 4.2 Implications for Clinical Practice

The findings from this review have identified that all health professionals have an important role in assessing and identifying social isolation and loneliness in people with CKD and kidney failure. Health organisations have minimal guidelines and protocols in predicting, managing and supporting people experiencing isolation and loneliness. The establishment of guidelines and protocols for all grades of kidney disease could potentially be introduced earlier within a CKD management plan as this review identifies that people need support and services. Additional support including kidney trained allied health staff could be effective for helping people navigate their illness trajectory and support not only the patient but their families as well. For renal nurses, they often develop a unique, therapeutic professional relationship with the patient, and further training in early detection of social isolation and loneliness may provide an opportunity for renal nurses to deliver early interventions or referral services. Nonetheless, the impact of social isolation and loneliness affects people negatively and makes CKD and kidney failure more challenging to effectively manage.

## 5 Conclusion

We found very few studies to include in this systematic review that focused on social isolation and loneliness. Several common aspects have emerged from the included studies that provide valuable insight into the impact that social isolation and loneliness have on people with CKD and kidney failure. Social isolation and loneliness affected psychological outcomes such as anxiety, depression and negative feelings that often made them question the point of continuing KRT. Different coping strategies and support from others to manage social isolation and loneliness were identified. Many people normalised social isolation and loneliness and accepted it as part of their disease that must be endured alone. Despite the advances in management and treatment for people living with CKD and kidney failure, social isolation and loneliness remain problematic for people with CKD and kidney failure, and support services are lacking to address this issue.

## Author Contributions

Amanda L. McKie was the principal leader and led the review process. Amanda L. McKie and Paul J. Buttel MScience drafted, reviewed and approved the manuscript.

## Funding

The authors received no specific funding for this work.

## Conflicts of Interest

The authors declare no conflicts of interest.

## Supporting information

Supplementary Table 1 MMAT.

Supporting information legend.

## Data Availability

As a systematic review, we relied solely on publicly published data. The authors can confirm that the data supporting the findings of this study are available within the paper and its supporting materials.

## References

[jorc70049-bib-0001] Almutary, H. , A. Bonner , and C. Douglas . 2016. “Which Patients With Chronic Kidney Disease Have the Greatest Symptom Burden? A Comparative Study of Advanced Ckd Stage and Dialysis Modality.” Journal of Renal Care 42, no. 2: 73–82. 26936486 10.1111/jorc.12152

[jorc70049-bib-0062] American Diabetes Association Professional Practice Committee . 2024. “11. Chronic Kidney Disease and Risk Management: Standards of Care in Diabetes—2024.” Supplement, Diabetes Care 47, no. S1: S219–S230. 10.2337/dc24-S011. 38078574 PMC10725805

[jorc70049-bib-0002] Asti, T. , M. Kara , G. Ipek , and B. Erci . 2006. “The Experiences of Loneliness, Depression, and Social Support of Turkish Patients With Continuous Ambulatory Peritoneal Dialysis and Their Caregivers.” Journal of Clinical Nursing 15, no. 4: 490–497. 16553763 10.1111/j.1365-2702.2006.01330.x

[jorc70049-bib-0003] Australian Institute of Health and Welfare (AIHW) . 2024. “Chronic Kidney Disease: Australian Facts.” https://www.aihw.gov.au/reports/chronic-kidney-disease/chronic-kidney-disease/contents/impact-of-chronic-kidney-disease/burden-of-chronic-kidney-disease.

[jorc70049-bib-0004] Barjaková, M. , A. Garnero , and B. D'hombres . 2023. “Risk Factors for Loneliness: A Literature Review.” Social Science & Medicine 334: 116163. 10.1016/j.socscimed.2023.116163. 37625251 PMC10523154

[jorc70049-bib-0005] Bártolo, A. , H. Sousa , O. Ribeiro , and D. Figueiredo . 2022. “Effectiveness of Psychosocial Interventions on the Burden and Quality of Life of Informal Caregivers of Hemodialysis Patients: A Systematic Review.” Disability and Rehabilitation 44, no. 26: 8176–8187. 10.1080/09638288.2021.2013961. 34913777

[jorc70049-bib-0006] Bikbov, B. , C. A. Purcell , A. S. Levey , et al. 2020. “Global, Regional, and National Burden of Chronic Kidney Disease, 1990–2017:A Systematic Analysis for the Global Burden of Disease Study 2017.” Lancet 395, no. 10225: 709–733. 32061315 10.1016/S0140-6736(20)30045-3 PMC7049905

[jorc70049-bib-0007] Christiansen, J. , P. Qualter , K. Friis , et al. 2021. “Associations of Loneliness and Social Isolation With Physical and Mental Health Among Adolescents and Young Adults.” Perspectives in public health 141, no. 4: 226–236. 34148462 10.1177/17579139211016077

[jorc70049-bib-0008] Chu, S. Y. , N. Ibrahim , N. Amit , et al. 2023. “Interventions to Reduce Caregiver Burden Among Caregivers of Chronic Kidney Disease (CKD) Patients: A Scoping Review.” Sage Open 13, no. 2: 21582440231178703. 10.1177/21582440231178703.

[jorc70049-bib-0009] De Silva, I. , N. Evangelidis , C. S. Hanson , et al. 2021. “Patient and Caregiver Perspectives on Sleep in Dialysis.” Journal of Sleep Research 30, no. 4: e13221. 33103303 10.1111/jsr.13221

[jorc70049-bib-0010] Diao, K. , J. Wang , and Y. Huang , et al. 2023. “The Experience of Social Isolation in Patients Receiving Peritoneal Dialysis: A Qualitative Study.” BMC Psychology 13: 947. 10.21203/rs.3.rs-3258674/v1. PMC12366228 40836246

[jorc70049-bib-0011] Driehuis, E. , I. Demirhan , W. S. Konijn , et al. 2025. “Determinants of Caregiver Burden Among Spouses of Patients With Kidney Failure: A Qualitative Study.” American Journal of Kidney Diseases 85, no. 4: 477–490.e1. 10.1053/j.ajkd.2024.11.005. 39788446

[jorc70049-bib-0012] Fakoya, O. A. , N. K. McCorry , and M. Donnelly . 2020. “Loneliness and Social Isolation Interventions for Older Adults: A Scoping Review of Reviews.” BMC Public Health 20, no. 1: 129. 32054474 10.1186/s12889-020-8251-6 PMC7020371

[jorc70049-bib-0013] Gerlach, L. B. , E. S. Solway , and P. N. Malani . 2024. “Social Isolation and Loneliness in Older Adults.” Journal of the American Medical Association 331, no. 23: 2058. 10.1001/jama.2024.3456. 38780951

[jorc70049-bib-0014] Goldman, N. , D. Khanna , M. L. El Asmar , P. Qualter , and A. El‐Osta . 2024. “Addressing Loneliness and Social Isolation in 52 Countries: A Scoping Review of National Policies.” BMC Public Health 24, no. 1: 1207. 10.1186/s12889-024-18370-8. 38693471 PMC11061917

[jorc70049-bib-0015] Hämmig, O. 2019. “Health Risks Associated With Social Isolation in General and in Young, Middle and Old Age.” PLoS One 14, no. 7: e0219663. 31318898 10.1371/journal.pone.0219663 PMC6638933

[jorc70049-bib-0016] Hoang, P. , J. A. King , S. Moore , et al. 2022. “Interventions Associated With Reduced Loneliness and Social Isolation in Older Adults: A Systematic Review and Meta‐Analysis.” JAMA Network Open 5, no. 10: e2236676. 10.1001/jamanetworkopen.2022.36676. 36251294 PMC9577679

[jorc70049-bib-0017] Holt‐Lunstad, J. , and A. Steptoe . 2022. “Social Isolation: An Underappreciated Determinant of Physical Health.” Current Opinion in Psychology 43: 232–237. 10.1016/j.copsyc.2021.07.012. 34438331

[jorc70049-bib-0018] Hong, Q. N. , S. Fàbregues , G. Bartlett , et al. 2018. “The Mixed Methods Appraisal Tool (MMAT) Version 2018 for Information Professionals and Researchers.” Education for Information 34, no. 4: 285–291. 10.3233/efi-180221.

[jorc70049-bib-0019] Hovadick, A. C. , V. R. Jardim , C. Paúl , A. Pagano , I. Reis , and H. Torres . 2021. “Interventions to Improve the Well‐Being of Family Caregivers of Patients on Hemodialysis and Peritoneal Dialysis: A Systematic Review.” PeerJ 9: e11713. 10.7717/peerj.11713. 34322322 PMC8300494

[jorc70049-bib-0020] Hui Joo, J. , A. Xie , and N. Choi , et al. 2025. “Loneliness, Self‐Efficacy and Adaptive Coping: Mixed Methods Analysis of Mediation in a Peer Support Intervention for Depression.” American Journal of Geriatric Psychiatry 33: 770–780. 10.1016/j.jagp.2025.02.013 PMC12085289 40121127

[jorc70049-bib-0021] Hwang, T.‐J. , K. Rabheru , C. Peisah , W. Reichman , and M. Ikeda . 2020. “Loneliness and Social Isolation During the COVID‐19 Pandemic.” International Psychogeriatrics 32, no. 10: 1217–1220. 32450943 10.1017/S1041610220000988 PMC7306546

[jorc70049-bib-0022] Iovino, P. , E. Vellone , N. Cedrone , and B. Riegel . 2023. “A Middle‐Range Theory of Social Isolation in Chronic Illness.” International Journal of Environmental Research and Public Health 20, no. 6: 4940. 36981849 10.3390/ijerph20064940 PMC10049704

[jorc70049-bib-0023] Jeong, D. , Y. J. Choi , and S. Sok . 2025. “Experience of Loneliness Among Middle‐Aged Hemodialysis Patients: Qualitative Study.” Journal of Nursing Management 2025, no. 1: 1013725. 10.1155/jonm/1013725. 40343257 PMC12061518

[jorc70049-bib-0024] Kalantar‐Zadeh, K. , T. H. Jafar , D. Nitsch , B. L. Neuen , and V. Perkovic . 2021. “Chronic Kidney Disease.” Lancet 398, no. 10302: 786–802. 34175022 10.1016/S0140-6736(21)00519-5

[jorc70049-bib-0025] Kerklaan, J. , E. Hannan , C. Hanson , et al. 2020. “Perspectives on Life Participation by Young Adults With Chronic Kidney Disease: An Interview Study.” BMJ Open 10, no. 10: e037840. 10.1136/bmjopen-2020-037840 PMC7569939 33067282

[jorc70049-bib-0026] Keskin, G. , A. Babacan Gümüş , and G. Taşdemir Yiğitoğlu . 2019. “Sexual Dysfunctions and Related Variables With Sexual Function in Patients Who Undergo Dialysis for Chronic Renal Failure.” Journal of Clinical Nursing 28, no. 1–2: 257–269. 10.1111/jocn.14602. 29968304

[jorc70049-bib-0027] Kidney Health Australia (KHA) . 2024. “Evidence Report Summary: 2021.” https://kidney.org.au/uploads/resources/KHA-Executive-Summary-Make-the-Link-Diabetes-Kidneys-and-Heart.pdf.

[jorc70049-bib-0028] Kirkeskov, L. , R. K. Carlsen , T. Lund , and N. H. Buus . 2021. “Employment of Patients With Kidney Failure Treated With Dialysis or Kidney Transplantation—A Systematic Review and Meta‐Analysis.” BMC Nephrology 22, no. 1: 348. 10.1186/s12882-021-02552-2. 34686138 PMC8532382

[jorc70049-bib-0029] Lee, B. O. , C. C. Lin , W. Chaboyer , C. L. Chiang , and C. C. Hung . 2007. “The Fatigue Experience of Haemodialysis Patients in Taiwan.” Journal of Clinical Nursing 16, no. 2: 407–413. 17239077 10.1111/j.1365-2702.2005.01409.x

[jorc70049-bib-0030] Lee, E. J. , A. K. Chang , and Y. C. Chung . 2021. “Socioecological Factors Affecting Fluid Restriction Adherence Among Korean Patients Receiving Hemodialysis: A Qualitative Study.” Journal of Transcultural Nursing 32, no. 3: 239–247. 32384013 10.1177/1043659620919162

[jorc70049-bib-0031] Levey, A. S. , K.‐U. Eckardt , N. M. Dorman , et al. 2020. “Nomenclature for Kidney Function and Disease: Report of a Kidney Disease: Improving Global Outcomes (KDIGO) Consensus Conference.” Kidney International 97, no. 6: 1117–1129. 10.1016/j.kint.2020.02.010. 32409237

[jorc70049-bib-0032] Luhmann, M. , S. Buecker , and M. Rüsberg . 2023. “Loneliness Across Time and Space.” Nature Reviews Psychology 2, no. 1: 9–23. 10.1038/s44159-022-00124-1. PMC9640887 36406179

[jorc70049-bib-0033] Malo, M.‐F. , A. Affdal , D. Blum , et al. 2022. “Lived Experiences of Patients Receiving Hemodialysis During the COVID‐19 Pandemic: A Qualitative Study From the Quebec Renal Network.” Kidney360 3, no. 6: 1057–1064. 35845331 10.34067/KID.0000182022 PMC9255873

[jorc70049-bib-0063] Manera, K. E. , B. J. Smith , K. B. Owen , P. Phongsavan , and M. H. Lim . 2022. “Psychometric Assessment of Scales For Measuring Loneliness and Social Isolation: An Analysis of the Household, Income and Labour Dynamics in Australia (HILDA) Survey.” Health and Quality of Life Outcomes 20, no. 1: 40. 35248075 10.1186/s12955-022-01946-6 PMC8897757

[jorc70049-bib-0034] Margono, H. M. 2024. “Dealing With Loneliness in Hemodialysis Patients: How to Prevent the Detrimental Effects of Loneliness.” Surabaya Psychiatry Journal/Jurnal Psikiatri Surabaya 13: 62–67.

[jorc70049-bib-0035] McKie, A. L. , and F. Gaida . 2022. “A Scoping Review of Spirituality and Religiosity in People Who Have Had a Kidney Transplant.” Nursing Open 9, no. 5: 2277–2288. 35670228 10.1002/nop2.1271 PMC9374409

[jorc70049-bib-0036] McKie, A. L. , M. Turner , and C. Paterson . 2023. “What Are the Qualitative Experiences of People Affected by Kidney Failure Receiving Haemodialysis?” Journal of Renal Care 49, no. 3: 170–190. 10.1111/jorc.12442. 36163591

[jorc70049-bib-0037] Miao, Y. , N. Jasim , C. Guha , et al. 2025. “Experiences of Loneliness and Social Isolation Among Young People With Chronic Physical Conditions: A Thematic Synthesis of Qualitative Studies.” Journal of Adolescence 97, no. 3: 593–608. 39550639 10.1002/jad.12445

[jorc70049-bib-0038] Mizumoto, J. , Y. Harada , and T. Terui , et al. 2024. “Identifying Unmet Social Needs in a Patient Living in Isolation: A Case Report.” Cureus 16, no. 1: e52429. 38371131 10.7759/cureus.52429 PMC10870694

[jorc70049-bib-0039] Motillon‐Toudic, C. , M. Walter , M. Séguin , J.‐D. Carrier , S. Berrouiguet , and C. Lemey . 2022. “Social Isolation and Suicide Risk: Literature Review and Perspectives.” European Psychiatry 65, no. 1: e65. 36216777 10.1192/j.eurpsy.2022.2320 PMC9641655

[jorc70049-bib-0064] Mushtaq, A. , and M. A. Khan . 2023. "Social Isolation, Loneliness, and Mental Health Among Older Adults During COVID‐19: A Scoping Review." Journal of Gerontological Social Work 67, no. 2: 143–156. 10.1080/01634372.2023.2237076. 37501381

[jorc70049-bib-0040] Nilsson, E. L. 2019. “Patients' Experiences of Initiating Unplanned Haemodialysis.” Journal of Renal Care 45, no. 3: 141–150. 10.1111/jorc.12282. 31317646

[jorc70049-bib-0041] O'Sullivan, R. , A. Burns , G. Leavey , et al. 2021. “Impact of the COVID‐19 Pandemic on Loneliness and Social Isolation: A Multi‐Country Study.” International Journal of Environmental Research and Public Health 18, no. 19: 9982. 34639283 10.3390/ijerph18199982 PMC8508181

[jorc70049-bib-0042] Page, M. J. , D. Moher , and P. M. Bossuyt , et al. 2021. “PRISMA 2020 Explanation and Elaboration: Updated Guidance and Exemplars for Reporting Systematic Reviews.” BMJ 372: n160. 33781993 10.1136/bmj.n160 PMC8005925

[jorc70049-bib-0043] Paterson, C. , M. Turner , M. E. Hooper , E. Ladbrook , L. Macauley , and A. Mckie . 2024. “Identifying Experiences of Supportive Care of Children and Young People Affected by Kidney Failure: A Qualitative Systematic Review.” Journal of Renal Care 50, no. 3: 252–274. 10.1111/jorc.12484. 38116998

[jorc70049-bib-0065] Pomeroy, M. L. , T. K. M. Cudjoe , A. E. Cuellar , et aI. 2023. “Association of Social Isolation With Hospitalization and Nursing Home Entry Among Community‐Dwelling Older Adults.” JAMA Internal Medicine 183, no. 9: 955. 10.1001/jamainternmed.2023.3064. 37486647 PMC10366946

[jorc70049-bib-0044] Riazuelo, H. 2021. “Couples Coping With the Serious Illness of One of the Partners.” Frontiers in Psychology 12: 638938. 33995193 10.3389/fpsyg.2021.638938 PMC8121455

[jorc70049-bib-0045] Rupp, S. , C. Fair , H. Korycinski , and M. Ferris . 2021. “‘It's What I Have, It's Not Who I Am’: a Qualitative Study of Social Support in Education/Employment Settings and Transition Readiness of Young Adults With End‐Stage Renal Disease.” International Journal of Environmental Research and Public Health 18, no. 12: 6596. 34205273 10.3390/ijerph18126596 PMC8296423

[jorc70049-bib-0046] Saedi, F. , M. Barkhordari‐Sharifabad , M. Javadi‐Estahbanati , and H. Fallahzadeh . 2019. “Sexual Function, Social Isolation, Loneliness and Self‐Esteem in Patients Undergoing Hemodialysis.” Sexuality & Disability 37, no. 3: 401–413. 10.1007/s11195-019-09575-6.

[jorc70049-bib-0047] Senteio, C. R. , and M. K. Ackerman . 2021. “Count Me Out: Perceptions of Black Patients Who Are on Dialysis but Who Are Not on a Transplant Waitlist.” Health Communication 1: 1–13. 10.1080/10410236.2021.1940017 34320893

[jorc70049-bib-0048] Shahgholian, N. , and H. Yousefi . 2018. “The Lived Experiences of Patients Undergoing Hemodialysis With the Concept of Care: A Phenomenological Study.” BMC Nephrology 19, no. 1: 338. 30477440 10.1186/s12882-018-1138-4 PMC6258413

[jorc70049-bib-0049] Shankar, R. , E. W. X. Lee , N. Luo , et al. 2025. “Assessing Caregiver Burden in Kidney Failure: A Systematic Review of Measurement Properties of Instruments.” Kidney Medicine 7, no. 9: 101054. 10.1016/j.xkme.2025.101054. 40791811 PMC12337670

[jorc70049-bib-0050] Sharma, S. , M. King , R. Mooney , et al. 2019. “How Do Patients From South Asian Backgrounds Experience Life on Haemodialysis in the UK? A Multicentre Qualitative Study.” BMJ Open 9, no. 5: e024739. 10.1136/bmjopen-2018-024739 PMC6530367 31101693

[jorc70049-bib-0051] Sluiter, A. , R. Cazzolli , A. Jaure , et al. 2024. “Experiences of Social Isolation and Loneliness in Chronic Kidney Disease: A Secondary Qualitative Analysis.” Clinical Journal of the American Society of Nephrology, 10.2215 19: 1405–1416. 39250223 10.2215/CJN.0000000000000529 PMC11556901

[jorc70049-bib-0052] Stern, C. , L. Lizarondo , J. Carrier , et al. 2020. “Methodological Guidance for the Conduct of Mixed Methods Systematic Reviews.” JBI Evidence Synthesis 18, no. 10: 2108–2118. 10.11124/jbisrir-d-19-00169. 32813460

[jorc70049-bib-0053] Suen, A. O. , A. S. Iyer , I. Cenzer , et al. 2023. “National Prevalence of Social Isolation and Loneliness in Adults With Chronic Obstructive Pulmonary Disease.” Annals of the American Thoracic Society 20, no. 12: 1709–1717. 37463307 10.1513/AnnalsATS.202304-288OC PMC10704233

[jorc70049-bib-0054] Sutherland, S. , K. E. Durley , K. Gillies , et al. 2021. “‘You See the Empty Bed Which Means It's Either a Transplant or a Death’: a Qualitative Study Exploring the Impact of Death in the Haemodialysis Community.” BMJ Open 11, no. 6: e046537. 10.1136/bmjopen-2020-046537 PMC8220525 34158299

[jorc70049-bib-0055] Thompson, K. N. , O. Oginni , J. Wertz , et al. 2025. “Social Isolation and Poor Mental Health in Young People: Testing Genetic and Environmental Influences in a Longitudinal Cohort Study.” European Child & Adolescent Psychiatry 34, no. 4: 1445–1455. 39259339 10.1007/s00787-024-02573-w PMC12000192

[jorc70049-bib-0056] Van Wilder, L. , P. Pype , F. Mertens , et al. 2021. “Living With a Chronic Disease: Insights From Patients With a Low Socioeconomic Status.” BMC Family Practice 22, no. 1: 233. 34789153 10.1186/s12875-021-01578-7 PMC8598397

[jorc70049-bib-0057] Williams, C. Y. K. , A. T. Townson , M. Kapur , et al. 2021. “Interventions to Reduce Social Isolation and Loneliness During COVID‐19 Physical Distancing Measures: A Rapid Systematic Review.” PLoS One 16, no. 2: e0247139. 33596273 10.1371/journal.pone.0247139 PMC7888614

[jorc70049-bib-0058] World Health Organization (WHO) . 2025. “Reducing the Burden of Noncommunicable Diseases Through the Promotion of Kidney Health and Strengthening Prevention and Control of Kidney Disease.” Executive board 156th Session. https://apps.who.int/gb/ebwha/pdf_files/EB156/B156_CONF6-en.pdf.

[jorc70049-bib-0059] Wu, B. 2020. “Social Isolation and Loneliness Among Older Adults in the Context of COVID‐19: a Global Challenge.” Global Health Research and Policy 5, no. 1: 27. 32514427 10.1186/s41256-020-00154-3 PMC7272234

[jorc70049-bib-0060] Yapa, H. E. , S. Chambers , L. Purtell , and A. Bonner . 2024. “Impact of Chronic Kidney Disease on Everyday Life: A Descriptive Qualitative Study.” Journal of Renal Care 50, no. 3: 201–211. 37573481 10.1111/jorc.12478

[jorc70049-bib-0061] Zou, J. , J. Xie , J. Zhang , H. Zhao , and P. Lu . 2024. “Coping Trajectory of Social Isolation in Individuals With Maintenance Haemodialysis: A Descriptive Qualitative Study.” International Journal of Nursing Studies Advances 6: 100193. 38746804 10.1016/j.ijnsa.2024.100193 PMC11080442

