# Supplementary material for: Social Isolation and Loneliness in People Living With Chronic Kidney Disease and Kidney Failure: A Mixed Method Systematic Review
Source: J Ren Care. 2026 Feb 7;52(1):e70049. doi: 10.1111/jorc.70049 (PMC12882110; doi:10.1111/jorc.70049)
Supplement: Supplementary file 1 — Supplementary Table 1 MMAT. [file JORC-52-0-s001.docx]

Supplementary Table 1: Mixed Methods Appraisal Tool, Hong et al (2018).

| Qualitative methodological quality criteria | | | | | |
| --- | --- | --- | --- | --- | --- |
| Author | 1.1 Is the qualitative approach appropriate to answer the research question? | 1.2 Are the qualitative data collection methods adequate to address the research question? | 1.3 Are the findings adequately derived from the data? | 1.4 Is the interpretation of results sufficiently substantiated by data? | 1.5 Is their coherence between qualitative data sources, collection, analysis and interpretation? |
| Diao et al. (2023) | Y | Y | Y | Y | Y |
| Jeong et al. (2025) | Y | Y | Y | Y | Y |
| Malo et al. (2022) | Y | Y | Y | Y | Y |
| Sluiter et al. (2024) | Y | Y | Y | Y | Y |
| Zou et al. (2023) | Y | Y | Y | Y | Y |
| Quantitative methodological quality criteria | | | | | |
| Author | 1.1 Is the sampling strategy relevant to address the research question? | 1.2 Is the sample representative of the target population? | 1.3 Are the measurements appropriate? | 1.4 Is the risk of nonresponse bias low? | 1.5 Is the statistical analysis appropriate to answer the research question? |
| Asti et al. (2006) | Y | Y | Y | CT | Y |
| Saedi et al. (2019) | Y | Y | Y | CT | Y |

Supplementary Table 1: The Mixed Methods Appraisal Tool was used to assess the quality of the studies according to Hong et al (2018). The abbreviations indicate Y; yes. N; no. CT; can’t tell.
