# Supplementary material for: Social Isolation and Loneliness in People Living With Chronic Kidney Disease and Kidney Failure: A Mixed Method Systematic Review
Source: J Ren Care. 2026 Feb 7;52(1):e70049. doi: 10.1111/jorc.70049 (PMC12882110; doi:10.1111/jorc.70049)
Supplement: Supplementary file 2 — Supporting information legend. [file JORC-52-0-s002.docx]

Figure 1

The Prisma flow diagram (Page et al., 2021) represents the screening process of the review. Twenty-four studies were identified through database screening and seven were included in the review.

Figure 2

Figure 2 identifies the four common aspects within the studies. Coping, support, psychological outcomes, and everyday life and routine where identified. The figure shows the studies that have the common aspects. Two of the studies identified all four aspects.

Supplementary Table 1

The Mixed Methods Appraisal Tool was used to assess the quality of the studies according to Hong et al (2018). The abbreviations indicate Y; yes. N; no. CT; can’t tell when assessing the study
